# Limits of the Ease-of-Retrieval Effect in Real and Fake News Credibility Judgments: Two Preregistered Experiments

**DOI:** 10.3390/bs16030327

**Published:** 2026-02-27

**Authors:** Ricardo M. Tamayo, Luis D. Ayala, Antonio Olivera-La Rosa

**Affiliations:** 1 Department of Psychology, Universidad Nacional de Colombia, Bogotá 11321, Colombia; rmtamayoos@unal.edu.co (R.M.T.); ldayalab@unal.edu.co (L.D.A.); 2 Basic and Applied Neuroscience Group, Department of Psychology, Universidad Católica Luis Amigó, Medellín 050034, Colombia; 3 Human Evolution and Cognition Group, University of the Balearic Islands, 07122 Palma, Spain

**Keywords:** misinformation, news credibility, retrieval fluency, familiarity

## Abstract

Ease-of-retrieval theories predict that information seems more credible when supporting reasons come to mind easily. However, it is unclear whether this holds for realistically ambiguous news headlines. We conducted two preregistered experiments (N = 128; N = 135) in which participants evaluated six pilot-tested real and fake headlines selected to minimize baseline credibility differences between veracity categories. In Experiment 1, participants generated either two or six reasons supporting or opposing each headline’s truth. In Experiment 2, the two-versus-six manipulation was crossed with a 20 s time limit. Headline veracity (real vs. fake) varied within participants, who then rated perceived task difficulty, credibility, and familiarity. Across experiments, generating six (vs. two) reasons increased perceived difficulty and reduced perceived deliberation time, indicating that the manipulation affected subjective fluency. However, linear mixed-effects analyses showed no reliable effect of the number of reasons on credibility. Credibility instead shifted with the direction of reasons required (supporting vs. opposing; ΔM ≈ 1.3) and increased with headline familiarity (r ≈ 0.40). These findings suggest that for ambiguous real-world headlines, classic ease-of-retrieval manipulations may alter perceived effort without translating into credibility judgments. Future work should test stronger fluency interventions and account for familiarity and motivational factors.

## 1. Introduction

The growing overlap between real and fake news makes it challenging to assess the truthfulness of ambiguous information. Important societal concerns such as political polarization and manipulation of public opinion are largely attributed to misinformation (Lefebvre et al., 2024; Lewandowsky et al., 2017; Roozenbeek et al., 2020). Although the exact extent of misinformation’s impact on society remains unclear (Adams et al., 2023; Miró-Llinares & Aguerri, 2023), understanding the cognitive mechanisms underlying judgments of credibility is crucial. In many real-world situations, individuals must quickly assess the credibility of news without explicit indicators of its truthfulness, often relying instead on intuitive judgments and subjective feelings. Among these metacognitive cues, ease of retrieval (EoR), that is, the subjective experience of ease or difficulty in retrieving relevant information (Schwarz et al., 1991), seems particularly pertinent, yet understudied in realistic contexts of news consumption.

The urgency of addressing social conflicts attributed to misinformation has drawn attention across disciplines (Lazer et al., 2018). Within psychology, research has examined cognitive (e.g., Schwarz & Jalbert, 2020) and motivational (e.g., Pennycook & Rand, 2020) factors. However, the specific influence of individuals’ interpretation of their subjective feelings of ease or difficulty as diagnostic information has not been fully investigated under conditions that foster motivated, effortful, and analytic processing—conditions that are often expected to reduce reliance on heuristic cues. This might be relevant for misinformation research because easy retrieval typically signals validity and increases reliance on retrieved content, whereas processing difficulty signals uncertainty and reduces reliance (Schwarz et al., 1991), and because previous studies have demonstrated EoR effects in various judgment contexts (Ask et al., 2012; Wänke, 2013). However, it is unclear whether these effects hold when individuals assess ambiguous, real-world headlines under conditions reflecting natural complexity and uncertainty. In other words, rather than assuming that ease of retrieval should generalize unchanged to realistic news evaluation, the present work treats ambiguity and high engagement as candidate boundary conditions under which classic EoR effects may weaken or disappear. Prior experiments tend to use less ambiguous materials or a single scenario or target, whereas we intend to assess multiple news headlines with real and fake uncertainty analogous to real scenarios, for instance in social media, where ambiguity characterizes encounters with news, where credibility judgments often involve rapid heuristic-driven decisions made under cognitive or temporal constraints.

Therefore, in the present study, we test whether the classic influence of subjective ease or difficulty in generating reasons translates to credibility judgments when evaluation is realistically ambiguous and likely to promote more effortful processing—that is, we test whether EoR remains diagnostic under boundary conditions that may attenuate heuristic use. The task withholds explicit information about headlines’ veracity, creating ambiguity similar to everyday news exposure in social media, with the potential to reveal the influence of EoR effects, thus contributing to refining both theoretical models of misinformation acceptance and practical strategies to mitigate susceptibility to fake news (Ross et al., 2019).

We conducted two preregistered experiments. Experiment 1 examined whether manipulating retrieval difficulty, by requiring participants to generate fewer (easier retrieval) or more (difficult retrieval) reasons, affects credibility judgments of ambiguous headlines. Experiment 2 extended this investigation by adding a time constraint, thereby simulating the rapid decision-making typical of real-world news evaluation. Since misinformation typically employs simpler and emotionally evocative content (Carrasco-Farré, 2022), we preregistered the canonical hypothesis that easier retrieval (fewer reasons) would increase perceived credibility because subjective ease can serve as a cue to validity in ambiguous contexts (Ask et al., 2012; Clore & Huntsinger, 2007; Schwarz & Jalbert, 2020). At the same time, because our procedure requires participants to generate reasons and make repeated evaluations, it may increase engagement and deliberation—conditions that can attenuate heuristic reliance—so the experiments also function as tests of boundary conditions for EoR in realistic headline evaluation.

Dual-process theories that distinguish intuitive (automatic and effortless) from deliberative (controlled and effortful) processing (Carruthers, 2012; De Neys, 2023; Evans, 2003; Kahneman et al., 2021) are commonly used to account for credibility judgments under uncertainty. Although people vary in their propensity to engage either mode when assessing credibility, intuitive processing tends to increase vulnerability to misinformation, whereas deliberative processing facilitates the identification of false content (Ross et al., 2019; Schwarz & Jalbert, 2020). Given the rapid and intuitive nature of information exposure online, many interventions explicitly target intuitive biases by encouraging deliberation (Lutzke et al., 2019; Pennycook et al., 2020, 2021). For instance, drawing individuals’ attention to accuracy can reduce misinformation sharing. Thus, understanding how cognitive feelings like EoR interact with intuitive and deliberative processes is relevant for such proposed interventions. Related work in communication research also links heuristic versus systematic processing to trust in media, providing converging support for considering processing mode in credibility judgments (Kaye & Johnson, 2021).

## 2. Feeling-as-Information Theory and Credibility Judgments

The Feeling-as-Information (FI) theory suggests that subjective feelings such as cognitive ease or difficulty provide heuristic cues influencing judgments and decisions (Song & Schwarz, 2008; Greifeneder et al., 2011). This theory differentiates between incidental feelings (unrelated to judgment targets) and integral feelings (directly related to processing relevant information) (Clore, 2017). EoR refers explicitly to integral feelings arising during the intentional recall or generation of relevant opinions (Schwarz et al., 1991; Wänke, 2013). For instance, individuals typically judge information as more credible when they experience ease in generating a few supporting reasons, interpreting retrieval fluency as a signal of higher truthfulness (Ask et al., 2012).

While familiarity (often increased through repetition) also impacts credibility judgments, known as the illusory truth effect (Dechêne et al., 2010; L. K. Fazio, 2020; Hasher et al., 1977; Udry & Barber, 2024; Unkelbach & Greifeneder, 2013), our research focuses specifically on the retrieval fluency dimension, examining how deliberate reasoning efforts shape credibility judgments, although we also measure the perceived familiarity of the materials. Understanding retrieval fluency might contribute directly to understanding how subjective cognitive feelings effectively guide judgment versus when other cognitive processes might dominate.

To ensure theoretical clarity, it is also essential to differentiate EoR clearly from priming effects (Higgins et al., 1977, 1985; Loersch & Payne, 2016), another source of cognitive accessibility influencing judgments. Priming involves semantic activation of content concepts independently of subjective cognitive feelings (Carroll & Slowiaczek, 1986; Fiedler, 2003; Fiedler et al., 2011; Payne et al., 2016). Importantly, previous evidence from EoR tasks aligns better with the Feeling-as-Information theory than with priming accounts. For example, if EoR effects stemmed primarily from semantic activation, asking participants to generate many reasons supporting a concept should enhance belief in that concept. However, studies show the opposite pattern: retrieval difficulty decreases judgments aligned with the primed concept, indicating subjective difficulty overrides semantic activation effects (Weingarten & Hutchinson, 2018). Thus, our study explicitly focuses on retrieval fluency (EoR), disentangling subjective cognitive feelings from mere content accessibility (priming effects).

## 3. The Current Studies and Hypotheses

The current experiments investigate how manipulating EoR impacts credibility judgments of difficult-to-discriminate real vs. fake news headlines. Derived from FI theory, we hypothesize that if participants treat retrieval fluency as diagnostic in this context, then when they can easily list a small number of reasons supporting why the headline might be **true**, the accompanying heightened processing fluency will lead them to rate the headline as more credible. In this situation, they rely on the heuristic “If it is easy to think of reasons why the headline is true, it is probably true.” Conversely, when generating supporting reasons is difficult because more reasons are required, fluency declines and credibility judgments should drop. Following the heuristic “If it is hard to think of reasons why the headline is true, it is probably false.”

We also included a reversed experimental condition in which participants listed reasons why the headline might be false. In this context, easily producing a few reasons for falsehood should increase fluency and decrease credibility, guided by the heuristic “If it is easy to think of reasons why the headline is false, it is probably false.” Conversely, when participants struggle to provide many reasons for falsehood, reduced fluency should raise credibility judgments, consistent with the heuristic “If it is hard to think of reasons why the headline is false, it is probably true.” Additionally, by introducing realistic conditions, ambiguity in Experiment 1 and time constraints in Experiment 2, we explicitly test whether EoR remains influential when judgments more closely resemble actual news evaluation contexts. In this sense, evidence for attenuated or null EoR effects would be theoretically informative by delineating conditions under which fluency cues are overshadowed by more engaged reasoning.

## 4. Experiment 1

### 4.1. Method

We preregistered all hypotheses, variables, exclusion criteria, and data analysis methods (https://aspredicted.org/54Y_8DK, accessed on 10 January 2026). Please refer to the Supplementary Materials Tables S1–S3 for raw data.

### 4.2. Design and Participants

We used a 2 (high and low accessibility) × 2 (true and false reasons) between-subjects factorial design. An a priori power analysis was conducted to determine the required sample size, targeting 80% power to detect an effect size of f = 0.25. This effect size is common in psychology (Brysbaert & Stevens, 2018) and aligns with previous similar research (Ask et al., 2012). The analysis indicated a need for 128 participants, with 32 participants per condition. Calculations were performed using G*Power software (Faul et al., 2009) and verified with the WebPower R package (Zhang & Yuan, 2018). Participants were randomly assigned to one of the four study conditions.

Participants were recruited via in-class announcements in first- and second-year psychology courses and completed the study in scheduled sessions in exchange for course credit. According to the preregistered exclusions, we removed four participants who failed the attentional check from further analyses. Additionally, we assessed data from two participants with unusually long completion times (greater than 1.5 interquartile ranges) to identify potentially poor-quality data (Leiner, 2019). However, after reviewing their data and finding their written explanations coherent and valid, we retained these participants. The final sample consisted of 128 participants, 52% female, aged 18–34 years (M = 21, SD = 2.96). The Supplementary Materials provide full descriptive statistics of participants’ demographics.

### 4.3. Materials

We used the Shiny package (Chang et al., 2021) in R to develop and run the experiment. First, we assembled a pool of 38 news headlines. Following a procedure similar to Pennycook et al. (2018), we sourced fake news from fact-checking websites and real news from traditional media outlets. The headlines mimicked the format commonly found on social networks such as Facebook, consisting of an image followed by a title and a brief description. An example of a stimulus (a fake headline) as presented to participants is provided in the Supplementary Materials (Figure S1).

We conducted a pilot test to ensure that the headlines used in the experiment were sufficiently ambiguous to challenge participants’ ability to distinguish between real and fake news under uncertainty. A pool of 38 fake and real headlines was rated for credibility on a nine-point scale (1 = not at all credible, 9 = very credible) by 36 university students (63.8% male) who were not part of the main experiment. We classified headlines as ambiguous if their mean credibility scores were close to the midpoint of the scale, indicating they were neither clearly real nor fake. Based on these ratings, we selected six headlines (three real and three fake) for the test trials.

### 4.4. Procedure

The experiment was conducted in a laboratory with individual cubicles to ensure privacy and focus attention. Upon arrival, participants completed the informed consent and a demographic survey. Then, we provided detailed instructions and administered a practice trial to familiarize them with the task. The instructions included an attentional control item, asking participants to “answer the question below with the first letter of the alphabet,” with options for the first four letters (A, B, C, D) provided at the bottom of the page.

Each trial began with the presentation of a news headline, displayed without a time limit. Participants rated whether they had previously encountered the headline on a 9-point Likert scale (1 = *I am sure I have not seen it*, 9 = *I am sure I have seen it*). Next, in line with Ask et al. (2012), participants were randomly assigned to one of four conditions to generate either two reasons (high accessibility) or six reasons (low accessibility) supporting or challenging the headline’s credibility. Although this two-versus-six approach is consistent with prior work, we did not additionally pilot-test these numbers in our specific context, so it is possible that both conditions felt relatively difficult given the ambiguous nature of the headlines. Importantly, participants did not know the headlines’ true veracity; “true reasons” and “false reasons” simply referred to whether they were supporting or opposing credibility, rather than reflecting actual knowledge of authenticity.

The instructions, prompts and the rating scales were also closely modelled after Ask et al. (2012). After each trial, participants rated the credibility of the news headline on a 9-point Likert scale (1 = not at all credible, 9 = very credible) for each of the six headlines. Participants then assessed difficulty using three items: (1) How difficult was it to generate the list of reasons? (2) How difficult would it have been to generate more reasons? (3) How difficult was it to find the last reason? Responses to each question were recorded on a 9-point Likert scale (1 = *very easy*, 9 = *very difficult*). Finally, participants rated their overall engagement with the task using a 9-point Likert scale ranging from 1 (*not at all engaged*) to 9 (*very engaged*).

### 4.5. Results

#### 4.5.1. Experimental Manipulation Check

We computed an aggregated difficulty score from the three difficulty-related questions, achieving strong internal reliability (Cronbach’s α = 0.80). The many-reasons group rated the task more difficult (Mdn = 8.00) than the few-reasons group (Mdn = 6.83), as confirmed by a Mann–Whitney U test (U = 3026, *p* < 0.001) conducted after detecting violations of normality assumptions (Shapiro–Wilk tests: many reasons W = 0.88, *p* < 0.001; few reasons W = 0.94, *p* = 0.003). Despite this statistical confirmation, we also observed that the median difficulty rating for the easy condition was relatively high (6.83 on a 9-point scale), meaning that participants probably experienced moderate versus very high difficulty rather than the classic easy versus difficult gap typically observed in previous EoR studies (e.g., Schwarz et al., 1991, Experiments 1 and 2). This reduced subjective contrast is a major limitation because it likely weakened the fluency signal that classic EoR effects depend on, reducing our ability to detect downstream effects on credibility judgments.

As expected, participants who generated many reasons took significantly more time (Mdn = 33.0 min) compared to those generating few reasons (Mdn = 18.0 min), also confirmed by a Mann–Whitney U test (U = 555.5, *p* < 0.001) following normality violations (Shapiro–Wilk: many reasons W = 0.93, *p* = 0.002; few reasons W = 0.92, *p* < 0.001). Participant engagement ratings were uniformly high (means ≥ 7 on a 9-point scale) across conditions, indicating strong task involvement and good data quality; however, such engagement also plausibly encouraged more analytic processing, which can attenuate reliance on fluency-based heuristic cues.

To evaluate task compliance, we further examined the written reasons provided. After removing stopwords, a word-frequency analysis showed that the most frequent terms corresponded to headline-relevant content, suggesting that participants engaged with the stimuli rather than responding randomly. In addition, reasons were coded into three categories: (a) content-based (references to visual or textual features of the headline), (b) factual (references to external knowledge or beliefs), and (c) no-content (nonsensical responses or statements such as “no reason”). No-content responses were rare (approximately 2–3%).

#### 4.5.2. Primary Results

We tested whether high perceived fluency or accessibility (two reasons) increased credibility ratings, and whether low perceived fluency or accessibility (many reasons) reduced credibility ratings. To this end, a linear mixed model (LMM) was used with participants as a random factor to account for the data’s hierarchical structure, reducing false positives and offering a robust alternative to traditional ANOVA (Meteyard & Davies, 2020).

The number of reasons did not have a significant effect on credibility ratings (b = 0.17, SE = 0.34, t(124) = 0.50, *p* = 0.62, d = 0.03). However, the type of reasons had a significant effect on credibility ratings (b = 1.37, SE = 0.34, t(124) = 4.08, *p* < 0.001, d = 1.4). More specifically, participants who provided reasons supporting the headlines’ veracity rated them as more credible (M = 4.92, SD = 1.41) than those who provided reasons refuting them (M = 3.65, SD = 1.25, d = 0.97). The interaction between the number and the type of reasons was also not significant (b = −0.19, SE = 0.48, t(124) = −0.39, *p* = 0.69, d = −0.03).

We checked the normality assumptions before running the main analysis. Both the Shapiro–Wilk test (W = 0.99, *p* < 0.001) and the Levene’s test (F(3, 764) = 5.12, *p* < 0.01) were significant. However, the residual plots indicated that the assumptions of linearity and independence were soundly met, considering that LMMs are robust to minor deviations from normality (Knief & Forstmeier, 2021), especially with sample sizes above 100 (N = 128 in our case).

**Figure 1:**
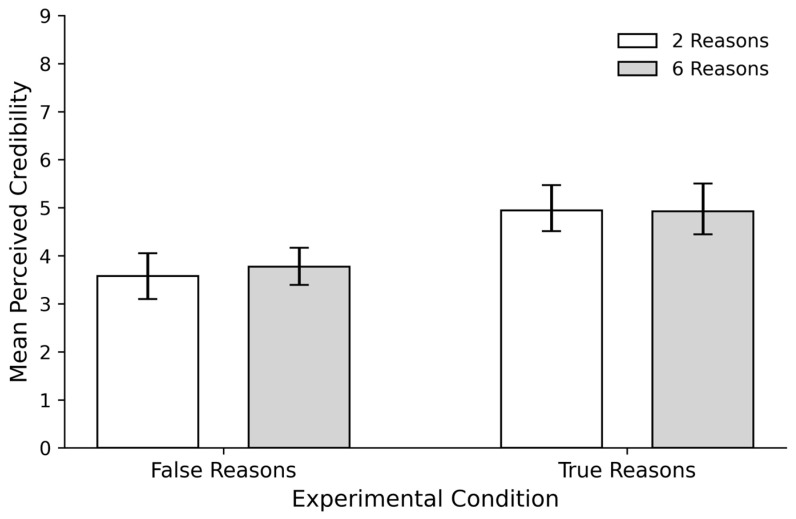
Mean Perceived Credibility by Type and Number of Reasons in Experiment 1. Error bars represent 95% confidence intervals (CIs) around the mean. *False Reasons:* participants to refute the headlines’ veracity. *True Reasons:* participants had to support the headlines’ veracity. The conditions *2 Reasons* and *6 Reasons* refer to the EoR manipulation, indicating the number of reasons participants were required to provide.

In sum, the ease or accessibility experienced when generating reasons (few vs. many) did not influence credibility judgments, while the type of reason provided by participants (supporting or refuting) aligned better with credibility (see Figure 1).

#### 4.5.3. Secondary Results

Based on the EoR manipulation, in our preregistration, we also hypothesized that the relationship between perceived difficulty and credibility ratings would differ between participants providing reasons supporting the headlines’ veracity and those providing reasons indicating the headlines’ falsehood. Consistent with the previous main results, Kendall’s tau correlations between perceived difficulty and credibility were not significant for participants providing true reasons (τ = −0.036, z = −0.388, *p* = 0.698) or false reasons (τ = 0.049, z = 0.520, *p* = 0.603), performed after detecting violations of normality assumptions (Shapiro–Wilk tests: true reasons, W = 0.940, *p* = 0.0038; false reasons, W = 0.883, *p* < 0.001), which also reject this secondary hypothesis.

In our pre-registration, we also aimed to analyze the role of the actual type of news (fake vs. real) on credibility. We conducted a three-way mixed-design ANOVA to examine the effects on credibility ratings, using the objective veracity of news headlines as a within-subjects factor, and the number and type of reasons participants provided (few vs. many and true vs. false) as between-subjects factors. The main effect of the number of reasons was not significant, F(1, 251) = 0.14, *p* = 0.71, η^2^ < 0.001, indicating that the number of reasons provided (few vs. many) did not significantly affect credibility ratings. The main effect of the type of reasons was significant, F(1, 251) = 42.44, *p* < 0.001, η^2^ = 0.14, suggesting again that participants who provided true reasons rated the headlines as significantly more credible than those who provided false reasons. The main effect of the type of news was not significant, F(1, 251) = 2.90, *p* = 0.09, η^2^ = 0.01. The interaction between the number of reasons and the type of reasons was not significant, F(1, 251) = 0.23, *p* = 0.63, η^2^ < 0.001. These results highlight the importance of the type of reasons in influencing credibility judgments and confirm that the actual veracity of the headlines did not significantly influence the main results.

To explore how familiarity modulates credibility, we also conducted a pre-registered ANCOVA with familiarity as the covariate and the number of reasons (few vs. many) and the type of reasons (true vs. false) as independent variables. Importantly, familiarity was significant, F(1, 123) = 14.29, *p* < 0.001, η^2^ = 0.10, meaning that higher credibility ratings were predicted by subjectively more familiar headlines. Consistent with previous analyses, the ANCOVA confirmed a significant main effect of the type of reasons, F(1, 123) = 16.13, *p* < 0.001, η^2^ = 0.12. The main effect of the number of reasons remained non-significant, F(1, 123) = 0.13, *p* = 0.72, η^2^ < 0.001, and the interaction between the number of reasons and the type of reasons was also not significant, F(1, 123) = 0.57, *p* = 0.45, η^2^ < 0.001. Assumptions of homogeneity of slopes (F(1, 120) = 0.66, *p* = 0.4188; F(1, 120) = 0.41, *p* = 0.5256; F(1, 120) = 1.44, *p* = 0.23) and homogeneity of variance (F(3, 124) = 0.8, *p* = 0.5) were met. Additionally, Pearson’s correlations confirmed that familiarity was positively associated with credibility ratings, r(126) = 0.40, *p* < 0.001, and this correlation held for both true reasons, r(62) = 0.40, *p* = 0.003, and false reasons, r(62) = 0.30, *p* = 0.04. These findings confirm the role of familiarity on credibility, highlighting the importance of prior exposure and cognitive fluency in shaping credibility judgments.

Finally, previous research has pointed out that some misinformation studies might conflate participants’ true ability to distinguish real from fake news with response bias (Batailler et al., 2022; Higham et al., 2024), so we carried out a supplemental non-preregistered analysis to address this potential issue. We employed a signal detection measure analogous to d-prime (*D*′), using participants’ ratings on a 9-point Likert scale, normalized by their standard deviations as in the following formula:

(1) $$D^{\prime }=\frac{{\bar{X}}_{Real}-{\bar{X}}_{Fake}}{\sqrt{s_{Real}^{2}+s_{Fake}^{2}}}$$

where ${\bar{X}}_{Real}$ is the mean credibility rating for real news, ${\bar{X}}_{Fake}$ the mean credibility for fake news, and the denominator contains the square root of the pooled standard deviations. However, this measure did not show statistical significance in a Kruskal–Wallis test for credibility ratings across experimental groups χ^2^(3) = 4.42, *p* = 0.22, conducted after observing violations of normality (W = 0.965, *p* = 0.002). Despite removing response bias by focusing on the detection of real vs. fake news, this analysis revealed that the experimental manipulation did not influence discriminability among the headlines.

### 4.6. Discussion

In this preregistered experiment, we examined whether EoR influenced credibility judgments of ambiguous news headlines. Consistent with Feeling-as-Information (FI) theory and prior research (Carrasco-Farré, 2022; Schwarz et al., 1991), we predicted that participants generating fewer reasons (high accessibility) would rate headlines as more credible than participants generating more reasons (low accessibility). Our design explicitly tested this by randomly assigning participants to one of four conditions formed by crossing the number of reasons (two vs. six) with the direction of reasoning (supporting vs. opposing headline credibility).

Contrary to our expectations, we found no significant effect of the EoR manipulation on credibility ratings. Instead, participants’ judgments were strongly influenced by whether they were asked to support or oppose the headline’s credibility: participants generating supportive reasons gave higher credibility ratings than those generating opposing reasons. This pattern emerged even though our manipulation check confirmed that providing six reasons was perceived as more difficult—and took longer—than providing two reasons. These null effects cannot be attributed to carry-over effects because each experimental group experienced only one condition, and there were no mixed factors involved in our experimental design.

A major design limitation concerns the context-sensitivity of EoR manipulations in this domain. Although we pilot-tested headlines to ensure they were sufficiently ambiguous, we did not pilot-test the classic manipulation of (two versus six reasons). Were the two reasons condition genuinely “easy” in this domain? Our manipulation check indicated that even two reasons felt moderately difficult, which narrowed the intended contrast between “easy” and “difficult” conditions. We only became aware of this narrower gap upon analyzing our data. However, the classic laboratory task (2 vs. 6 reasons) reliably yields a pronounced contrast, whereas the realistically ambiguous headlines here appear to have made even two reasons feel challenging.

Our results also differ notably from those of Ask et al. (2012), who observed EoR effects on credibility judgments. While we closely replicated key aspects such as the number of reasons, instructions, sample size, and manipulation checks, our task was arguably more complex because Ask et al. focused on a single judgment scenario (evaluating a videotaped candidate’s believability), whereas we required participants to assess multiple ambiguous headlines. On one hand, evaluating multiple headlines may improve measurement reliability, but on the other, it can increase participants’ engagement and promote more deliberate, analytical processing, thereby overriding heuristic reliance on cognitive fluency cues (Greifeneder et al., 2011; Schwarz et al., 1991). In fact, in line with prior research on familiarity (Bronstein et al., 2021; Olson & Fazio, 2008), our secondary analyses showed that subjective familiarity with the headlines enhanced perceived credibility independently of retrieval fluency.

Demand characteristics may also have contributed to the null effect. Participants who were explicitly requested to generate reasons supporting or opposing each headline might have inferred that researchers expected their subsequent credibility ratings to align with that stance, diminishing any influence from subjective ease. Because credibility was rated immediately after generating reasons, participants may also have treated their just-produced arguments as direct inputs to the judgment, or aimed to remain consistent with the assigned stance. This sequencing likely amplified the direction-of-reasons effect and reduced sensitivity to more subtle fluency-based (EoR) cues. Additionally, because our headlines were deliberately chosen to minimize obvious truthfulness cues, participants may have felt compelled to adopt more careful, effortful reasoning rather than relying on fluency-based heuristics.

In summary, we found no evidence of EoR effects on credibility judgments under realistic, cognitively demanding conditions reminiscent of evaluating real vs. fake news on social media. Our findings imply that EoR might be constrained by heightened task complexity, ambiguity, participant engagement, and possible demand characteristics. Notably, having discovered that the difference between “2 vs. 6” reasons was narrower than anticipated, we introduced time pressure in a second experiment in hopes of increasing reliance on intuitive processing. According to dual-process models like the MODE model (Groncki et al., 2021), subjective cognitive cues (e.g., retrieval fluency) should dominate when people have less opportunity for systematic thought. Therefore, Experiment 2 was designed to impose strict time constraints, aiming to create an environment where EoR might more clearly influence credibility judgments.

## 5. Experiment 2

We designed a second experiment to enhance the likelihood that the EoR manipulation would influence credibility judgments. Consequently, we introduced time pressure, which can significantly influence how individuals evaluate the credibility of information by making them more reliant on intuitive heuristics, such as ease of processing, and less likely to engage in systematic analytical thinking (Kahneman, 2011). This manipulation also provides practical ecological validity by simulating the fast-paced evaluation of news headlines on social media, where individuals often make quick, superficial credibility judgments amidst information overload.

We hypothesized that the absence of time pressure in our first experiment might have contributed to the deviation from results observed in other studies on ease-of-processing manipulations. Specifically, when the informational value of feelings is low, these feelings are less likely to influence judgments and decisions (Greifeneder et al., 2011). High processing investment, requiring cognitive resources such as attention, memory and abstraction, tends to decrease the influence of intuitive feelings (Kahneman, 2011; Schwarz & Jalbert, 2020). For instance, according to the MODE model (R. H. Fazio, 1990), the extent to which intuitive processing influences judgments is determined by the motivation and opportunity to process information deliberately. When motivation and opportunity for deliberation are high, judgments are more likely to be analytical (Kahneman, 2011).

In Experiment 2, we asked whether limiting the opportunity for deliberative processing by introducing time pressure would allow feelings of processing to affect credibility judgments more directly. We expected that the time constraint would lead participants to rely more on heuristic cues, potentially revealing effects that were masked by high processing engagement and great difficulty in the previous experiment. Since Experiment 1 did not show a significant interaction between the type of reason (supporting or refuting) and the ease of processing on credibility judgments, and because the focus of the present paper is on predictions from the FI theory, we removed the type of reason factor to concentrate on the effects of time constraint and ease of processing.

### 5.1. Method

Experiment 2 is a direct theoretical follow-up to Experiment 1. As in the first study, we preregistered the research before starting data collection (https://aspredicted.org/DHG_C5W, accessed on 10 January 2026).

### 5.2. Participants and Design

We focused on the primary variables of interest—ease of processing and time restriction—by excluding the type-of-reason factor. This simplification resulted in a 2 (high vs. low accessibility) × 2 (with vs. without time restriction) between-subjects factorial design. We calculated the sample size a priori to be 128 participants, following the same approach as in Experiment 1. However, we collected data from 135 participants recruited using the same procedures as in Experiment 1. To account for potential issues such as computer crashes or data corruption, we slightly oversampled. The final sample was 63% female, with ages ranging from 17 to 52 years (M = 21, SD = 4.03). No participants overlapped across experiments. Full descriptive statistics are reported in the Supplementary Materials.

### 5.3. Materials and Procedure

The materials (news headlines), apparatus (Shiny App), methods, and procedure closely followed those of the first study. The news headlines were displayed for at least 30 s. Participants were prompted to read and observe the headlines carefully before continuing. Subsequently, all participants were required to write reasons (2 or 6, depending on the condition) explaining why the headline might be true. In the time-constrained conditions, participants had only 20 s per reason (resulting in a total of 40 s for the 2-reason condition and 120 s for the 6-reason condition).

When the time had expired or when all the requested reasons had been written, participants rated the credibility of the headline using a Likert scale similar to the one used in the first experiment. Finally, participants answered the same questions about difficulty and engagement as before. Additionally, we added a new manipulation check adapted to the new time-constrained condition: Did you have enough time to think and write the reasons? This question was rated on a scale from 1 (not at all) to 9 (plenty of time).

### 5.4. Results

#### 5.4.1. Manipulation Check

Similar to the first study, we averaged the scores of the perceived difficulty questions (Cronbach’s α = 0.73). Since the time constraint can influence perceived difficulty, we compared these scores across experimental conditions. Participants in the time-constrained conditions found the task more difficult when they had to report six reasons (*Mdn* = 7.67) compared to those who reported two reasons (*Mdn* = 6.67), *U* = 317, *p* < 0.01. A similar pattern emerged in the conditions without time constraints, where participants who reported six reasons also found the task more difficult (*Mdn* = 8.00) compared to those who reported two reasons (*Mdn* = 6.67), *U* = 224, *p* < 0.01.

Additionally, we evaluated the effectiveness of the time pressure manipulation with the question, “Did you have enough time to think and write the reasons?” Participants in the conditions without time constraints reported having sufficient time (*Mdn* = 8.00), whereas those in the time-constrained conditions reported having less time (*Mdn* = 5.00), *U* = 829, *p* < 0.01.

#### 5.4.2. Primary Results

**Figure 2:**
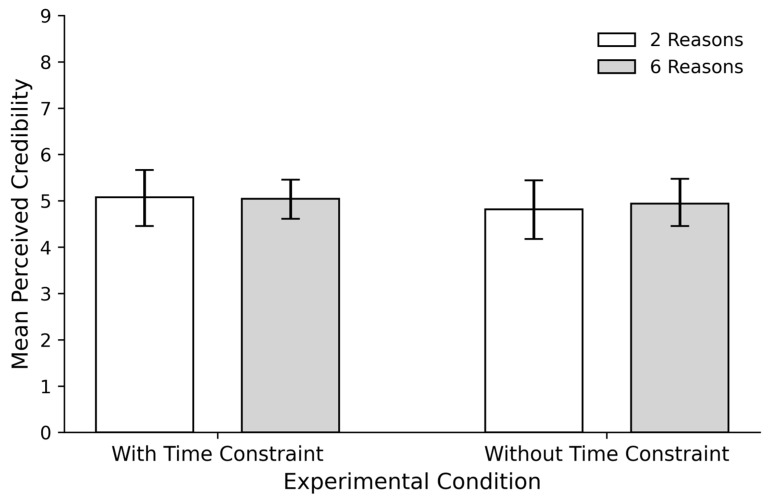
Mean Perceived Credibility by Type and Number of Reasons in Experiment 2. Error bars represent 95% confidence intervals (CIs) around the mean. *Time restriction* indicates the condition where participants experienced a limited time to provide reasons, while *No time restriction* indicates the condition where participants had no time limit. The conditions *2 Reasons* and *6 Reasons* refer to the EoR manipulation, indicating the number of reasons participants were required to provide.

As in the first study, we used an LMM with participants as a random factor. The main effects of number of reasons (β = −0.03, *SE* = 0.29, *t* = 0.09, *p* = 0.92) and temporal restriction (β = −0.43, *SE* = 0.29, *t* = 1.47, *p* = 0.14) were not significant. Similarly, the interaction between the main factors (β = 0.24, *SE* = 0.42, *t* = 0.58, *p* = 0.56) was not statistically significant. That is, no differences in credibility scores were observed between any of the experimental conditions (see Figure 2).

### 5.5. Discussion

In this second experiment, we introduced time pressure to determine whether limiting deliberation might reveal EoR effects that were masked in Experiment 1. Specifically, we anticipated that forcing participants to respond quickly—by restricting the time available to generate reasons—would encourage greater reliance on heuristic cues, consistent with the MODE model (R. H. Fazio, 1990). We also wanted to see whether the relatively small subjective gap between “2 reasons” and “6 reasons” observed in Experiment 1 would persist when participants were under time constraints.

Contrary to our expectations, neither the number of reasons provided nor the presence of time pressure reliably influenced credibility ratings. Even though participants in the time-constrained condition reported having less time to think and found generating six reasons more difficult, this did not translate into higher credibility ratings for headlines in the ostensibly “easy” (two-reason) condition. As in Experiment 1, the manipulation of accessibility was statistically valid—participants perceived a difference between “2 vs. 6 reasons”—yet it did not affect headline credibility. These findings indicate that time pressure alone was insufficient to elicit EoR effects, potentially due to persistent contextual or motivational factors.

## 6. General Discussion

In this study, we tested whether varying the ease of retrieving reasons to trust or distrust ambiguous headlines would shape credibility judgments of real and fake news. Across two preregistered experiments, participants who generated fewer (“easy”) vs. more (“difficult”) reasons did not differ in their credibility ratings. There were no discernible EoR effects, not even from a time pressure modification meant to promote heuristic processing. These results provide boundary conditions under which the EoR phenomenon may be diminished, rather than refuting it. The subjective distinction between “easy” and “difficult” retrieval may become far less noticeable in genuine, ambiguous situations where participants are encouraged to think critically and news items provide few clear truth indications. Consequently, cognitive fluency cues can be easily overshadowed by deeper analytical engagement, strong motivations, or demand characteristics. In particular, requiring participants to generate written reasons for multiple ambiguous headlines in a laboratory setting may shift the judgment context away from low-effort news browsing and toward deliberative, consistency-oriented evaluation—precisely the conditions under which fluency cues are most likely to be discounted.

### 6.1. Methodological Considerations

We were concerned about whether insufficient power masked an otherwise reliable effect. To address this, we combined data from participants in both experiments who completed identical tasks (generating two vs. six reasons, without time pressure). This nearly doubled the sample size, providing adequate power (86%) to detect a medium effect (d ≈ 0.53). However, we still observed no meaningful differences in credibility judgments (d = 0.08). Hence, statistical power alone is unlikely to explain our null effects.

A major limitation involves how “easy” and “difficult” manipulations function in the complex domain of news credibility. Although our manipulation checks showed that participants in “many reasons” conditions reported significantly greater difficulty, the “few reasons” condition still felt subjectively challenging. When facing inherently ambiguous headlines, even an ostensibly small retrieval task may demand substantial cognitive effort, compressing the distinction between easy and difficult retrieval. Careful pilot testing must therefore verify not only that the headlines are ambiguous, but also that the target contrast in difficulty (e.g., two vs. six reasons) truly maps onto participants’ subjective experiences.

A second explanation involves participants’ motivation and analytical engagement. Generating reasons for credibility assessments, especially in a laboratory setting that emphasizes thoroughness, can encourage deliberative processing. Prior research (Bronstein et al., 2021) suggests that highly engaged participants, especially university students, rely less on heuristic fluency cues if they have sufficient cognitive resources to think critically. Similarly, motivational factors (such as social identification or personal convictions) may take precedence over ease of retrieval. Simply changing the number of reasons might not significantly change participants’ final assessments if they are already prepared to examine ambiguous headlines.

We did not include an individual-differences measure such as Need for Cognition (NFC), which could moderate the extent to which participants spontaneously engage in deliberative processing during credibility evaluation. Future studies could test whether EoR effects emerge more reliably among low-NFC participants, or whether high-NFC participants are particularly likely to override fluency cues with analytic reasoning.

Notably, we did not see the expected impact on credibility even in Experiment 2, where time constraints were supposed to promote intuitive over deliberate reasoning. Participants may have adapted by producing fewer, stronger arguments under time pressure, which would have allowed them to maintain an analytical approach even with shorter deadlines, consistent with dual-process theories such as the MODE model (R. H. Fazio, 1990; Olson & Fazio, 2008).

Demand characteristics are likely a major interpretive factor in our experiments. When participants are explicitly instructed to generate reasons supporting or challenging a headline’s credibility, they may infer that their subsequent ratings “should” match the reasons they just produced, thereby overshadowing any subtle fluency-based cues. This concern is heightened by the sequence in which reason generation is immediately followed by a credibility rating, which makes alignment with the generated reasons especially salient. We nonetheless adopted this procedure because earlier work suggested that such demand effects do not always dominate when retrieval difficulty is manipulated effectively (Ask et al., 2012). In our data, however, participants’ credibility ratings still aligned strongly with the direction of the reasons they generated, independent of the experienced ease or difficulty of retrieval.

These issues could be addressed in future studies by employing cover stories, including filler tasks, or measuring credibility indirectly, which would lessen the likelihood that participants will intentionally align their credibility judgments with the type of reasons they provided. In order to ascertain whether demand characteristics were involved, researchers could also evaluate participants’ awareness of the study hypotheses after the experiment.

### 6.2. Implications

Although our null results resonate with recent large-scale replication efforts (Groncki et al., 2021) that also failed to detect classic EoR effects, even in more controlled settings reminiscent of Schwarz et al. (1991), our replication failures highlight methodological constrains such as question order (especially eliciting reasons before credibility ratings), number of retrieval items, timing, and instruction style, that can affect whether fluency cues surface in judgment. The present research suggests that EoR may have reduced applicability in complex, real-world tasks. Ambiguous content, high participant engagement, and motivational factors can all undermine or mask fluency cues. Rather than invalidating the principle that subjective feelings can shape judgments, our findings help delineate situations where they are less likely to exert a measurable effect.

Theoretically, these results imply that in challenging or ambiguous contexts, even “few reasons” feel effortful for participants and that credibility judgments may rest more on motivational states than on heuristic feelings of difficulty. While EoR remains robust in many controlled laboratory paradigms, in practice, interventions designed to counter misinformation may be better served by approaches that actively encourage critical reasoning and deliberate argument evaluation. Media literacy training that emphasizes systematic source scrutiny and more reflective forms of judgment could increase resilience to misinformation more effectively than interventions relying on manipulation of subjective fluency cues because, as our results demonstrate, such manipulations require high subjective contrasts between easy and difficult conditions.

In sum, our findings expand current knowledge on EoR by identifying specific conditions under which it may fail to influence credibility judgments: realistic news contexts characterized by ambiguity, motivated processing, and potentially powerful demand characteristics.

Future studies should investigate whether greater difficulty contrasts (e.g., 1 vs. 6 reasons) or alternative designs (e.g., hidden hypotheses, indirect credibility measures) might boost the impact of EoR. Researchers could also evaluate the quality of generated arguments, measure participants’ motivations directly, and further explore how personal or emotional investment interacts with fluency cues. By systematically addressing these conditions, we can refine theoretical models of how people judge the credibility of uncertain information and develop more effective interventions for countering misinformation in the real world.

## Data Availability

The anonymized datasets and analysis code supporting the findings of this study are publicly available in the Open Science Framework repository at https://doi.org/10.17605/OSF.IO/6AU75.

## Supplementary Materials

The following supporting information can be downloaded at: https://www.mdpi.com/article/10.3390/bs16030327/s1, Table S1: Demographic characteristics—Experiment 1; Table S2: Demographic characteristics—Experiment 2; Table S3: Headlines used in Experiments 1 and 2 (Spanish-language stimuli, including practice headline); Figure S1: Example of a fake news headline stimulus as presented to participants.
