# Supplementary material for: Limits of the Ease-of-Retrieval Effect in Real and Fake News Credibility Judgments: Two Preregistered Experiments
Source: Behav Sci (Basel). 2026 Feb 27;16(3):327. doi: 10.3390/bs16030327 (PMC13023825; doi:10.3390/bs16030327)
Supplement: Supplementary file 1 [file behavsci-16-00327-s001.zip › behavsci-4128135-supplementary.pdf]

Supplementary Materials

Limits of the ease-of-retrieval effect in real and fake news credibility judgments: Two preregistered experiments

This file contains (1) demographic summaries for Experiments 1 and 2; (2) the list of Spanish-language headlines used as stimuli (including the practice headline); and (3) one example fake headline stimulus provided to illustrate the experimental materials.

Table S1. Demographic characteristics – Experiment 1 (N = 128)

VARIABLE	LEVEL	TOTAL	[1]GROUP 1	[2]GROUP 2	[3]GROUP 3	[4]GROUP 4
**SEX %**	Female	52.34	13.28	10.94	12.50	15.62
	Male	45.32	10.94	13.28	11.72	9.38
	Other	2.34	0.78	0.78	0.78	—
**EDUCATION %**	High-school	64.06	16.41	16.41	15.62	15.62
	Technical	12.50	4.69	2.34	3.91	1.56
	Undergraduate	23.44	3.91	6.25	5.47	7.81
**SES %**	1 (Lowest)	11.71	3.12	2.34	3.91	2.34
	2	30.47	6.25	10.16	6.25	7.81
	3	44.54	10.16	9.38	13.28	11.72
	4	13.27	5.47	3.12	1.56	3.12
**AGE, MEAN (SD)**	—	21.42 (2.96)	21.19 (2.63)	21.59 (3.19)	21.78 (2.38)	21.12 (3.59)

Group 1: 6 reasons, oppose credibility *(n = 32)*
Group 2: 2 reasons, oppose credibility *(n = 32)*
Group 3: 6 reasons, support credibility *(n = 32)*
Group 4: 2 reasons, support credibility *(n = 32)*

Table S2. Demographic characteristics – Experiment 2 (N = 135)

VARIABLE	LEVEL	TOTAL	[1]GROUP 1	[2]GROUP 2	[3]GROUP 3	[4]GROUP 4
**SEX %**	Female	63.00	11.85	18.52	16.30	16.30
	Male	35.56	13.33	5.93	6.67	9.63
	Other	1.48	0.74	0.74	—	—
**EDUCATION %**	High-school	71.11	19.26	19.26	15.56	17.04
	Technical	16.99	2.22	2.22	5.19	7.41
	Undergraduate	11.85	4.44	3.70	2.22	1.48
**SES %**	1 (Lowest)	9.63	0.74	4.44	2.22	2.22
	2	30.37	7.41	5.93	7.41	9.63
	3	34.07	9.63	8.89	7.41	8.15
	4	23.70	8.15	5.19	5.19	5.19
	5 (Highest)	2.22	—	0.74	0.74	0.74
**AGE, MEAN (SD)**	—	20.90 (4.04)	20.34 (1.83)	20.47 (2.93)	22.00 (6.28)	20.91 (4.02)

Group 1: 2 reasons, no time-limit *(n = 35)*
Group 2: 6 reasons, no time-limit *(n = 34)*
Group 3: 2 reasons, 20 s limit *(n = 31)*
Group 4: 6 reasons, 20 s limit *(n = 35)*

Table S3. Headlines used in Experiments 1 and 2

ID	Veracity	Use	Headline (Spanish)
H1	Real	Experimental	Casi 3.000 casos ha abierto Procuraduría por mal manejo de la pandemia
H2	Real	Experimental	Sanciones y castigos alrededor de COVID-19, una tendencia mundial
H3	Real	Experimental	Unisimón participa en el desarrollo de una potencial vacuna contra el covid-19
H4	Real	Practice	Kawasaki, la rara enfermedad relacionada con covid-19
H5	Fake	Experimental	El uso compulsivo de mascarillas ocasiona la propagación del hongo infeccioso "Candid Auris" en Florida
H6	Fake	Experimental	Colombianos esperan el camión de las vacunas a las afueras del Aeropuerto El Dorado
H7	Fake	Experimental	Noruega reporta muertes de octogenarios por vacunas Covid-19

Note. All headlines were presented in Spanish, as the study was conducted with Spanish-speaking participants. Headline H4 was used only for the practice trial.

Figure S1. Example of a fake news headline stimulus as presented to participants.

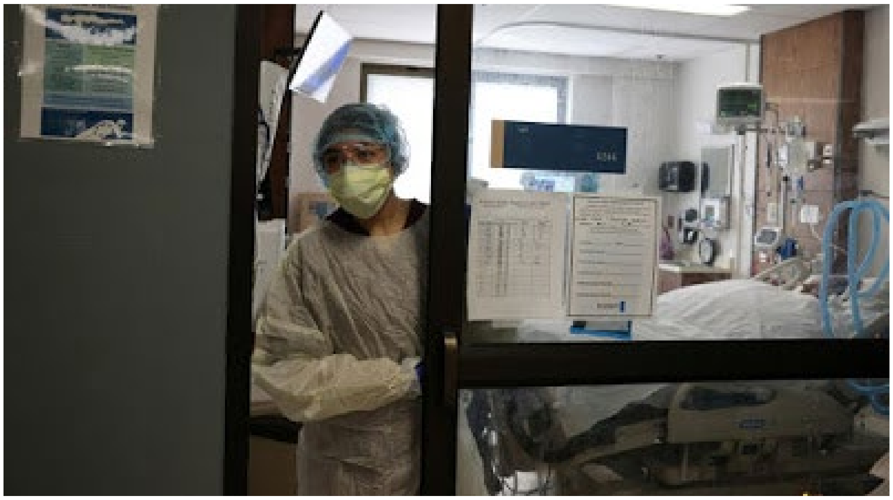

Compulsive mask use linked to spread of infectious fungus “Candida auris” in Florida

Last summer, about thirty patients with COVID-19 who were admitted to a specialized ward in a Florida hospital became infected with the fungus Candida auris, according to a study released Friday by the U.S. Centers for Disease Control and Prevention (CDC).
